# Can harmful lifestyle, obesity and weight changes increase the risk of breast cancer in BRCA 1 and BRCA 2 mutation carriers? A Mini review

**DOI:** 10.1186/s13053-021-00199-6

**Published:** 2021-10-27

**Authors:** A. Daniele, R. Divella, B. Pilato, S. Tommasi, P. Pasanisi, M. Patruno, M. Digennaro, C. Minoia, M. Dellino, S. Pisconti, P. Casamassima, E. Savino, A. V. Paradiso

**Affiliations:** 1 Experimental Oncology and Biobank Management Unit, IRCCS Istituto Tumori “Giovanni Paolo II” Bari, Viale Orazio Flacco 65, 70124 Bari, Italy; 2 Molecular Diagnostics and Pharmacogenetics Unit, IRCCS Istituto Tumori “Giovanni Paolo II” Bari, Bari, Italy; 3 Epidemiology and Prevention Unit, Fondazione IRCCS Istituto Nazionale dei Tumori di Milano, Milan, Italy; 4 Experimental Oncology, Center for Study of Heredo-Familial Tumors, IRCCS- Istituto “Tumori “Giovanni Paolo II”, Bari, Italy; 5 Hematology Unit, IRCCS Istituto Tumori “Giovanni Paolo II” Bari, Bari, Italy; 6 Gynecologic Oncology Unit, IRCCS Istituto Tumori “Giovanni Paolo II” Bari, Bari, Italy; 7 Medical Oncology Unit, Ospedale Moscati, Taranto, Italy; 8 Clinical Pathology Laboratory Unit, IRCCS Istituto Tumori “Giovanni Paolo II” Bari, Bari, Italy; 9 Science Direction, IRCCS Istituto Tumori “Giovanni Paolo II” Bari, Bari, Italy

**Keywords:** BRCA mutation, Lifestyle, Obesity, BRCA-associated cancer

## Abstract

**Background and aim:**

The BRCA 1 and BRCA 2 genes are associated with an inherited susceptibility to breast cancer with a cumulative risk of 60% in BRCA 1 mutation carriers and of 30% in BRCA 2 mutation carriers. Several lifestyle factors could play a role in determining an individual’s risk of breast cancer. Obesity, changes in body size or unhealthy lifestyle habits such as smoking, alcohol consumption and physical inactivity have been evaluated as possible determinants of breast cancer risk. The aim of this study was to explore the current understanding of the role of harmful lifestyle and obesity or weight change in the development of breast cancer in female carriers of BRCA 1/2 mutations.

**Methods:**

Articles were identified from MEDLINE in October 2020 utilizing related keywords; they were then read and notes, study participants, measures, data analysis and results were used to write this review.

**Results:**

Studies with very large case series have been carried out but only few of them have shown consistent results. Additional research would be beneficial to better determine the actual role and impact of such factors.

## Introduction

Breast cancer (BC) is the most common female malignancy worldwide. Approximately 5–10% of breast cancer cases are hereditary and arise from autosomal dominant mutations in specific cancer genes, including the two breast cancer susceptibility genes BRCA 1 and BRCA 2. Women who carry these mutations have up to an 80% risk of developing breast cancer [1–3]. Identifying modifiable exposures is very important in BRCA 1/2 mutation carriers. The onset of breast cancer in these women may be influenced by genetic factors such as AdipoQ gene polymorphism associated to alterations in adipokines [4–6]. Evidence suggests that additional modifying factors influence cancer penetrance in BRCA 1/2 mutations carriers. Exposure to environmental factors and unhealthy lifestyle factors, including obesity, change in body size, smoking, alcohol consumption and physical inactivity, have been suggested to increase breast cancer (BC) risk in BRCA 1/2 mutation carriers [7–9]. An association of these factors has been widely reported to enhance the risk of developing cancer [10]. In a previous study of 2020 (Bruno et al.), we examined the relationships between selected lifestyle, metabolic exposures and BRCA related cancer in 502 women with BRCA mutations and found that increased fat mass and dysmetabolism were significantly associated with BC risk and had a greater effect in BRCA 2 positive women [11]. Obesity may increase BC risk through multiple mechanisms including insulin-resistance, metabolic syndrome, increased production of sex hormones and insulin-like growth factor-1 (IGF-1). In 2020, Pasanisi et al. reported that a Mediterranean diet with protein restriction is effective in reducing potential modulators of BRCA penetrance [12].

Given the high penetrance of BRCA 1 and BRCA 2 mutations, prevention and lifestyle changes have an extremely important risk-reducing role in women who have a higher risk of developing breast cancer.

## Methods

**Fig. 1:**
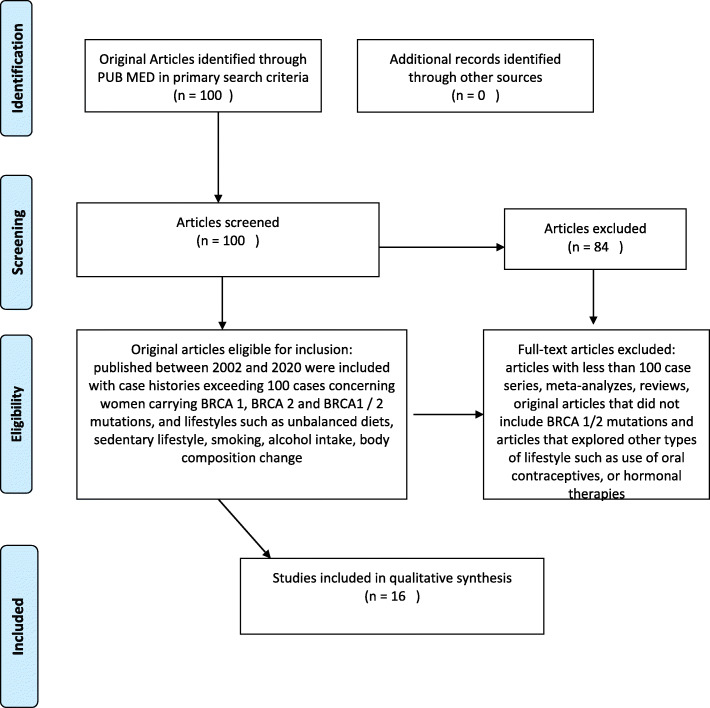
Strategy used to identify literature for review

A broad review of the literature was carried out using MEDLINE (via PubMed) and sixteen articles published from 2002 to 2020, were selected from a total of one hundred. Search terms included keywords, combining the conditions (BRCA 1, BRCA 2, mutations, carriers, breast cancer risk), obesity, change in body weight and lifestyle (alcohol, smoking, physical inactivity). We included only original peer-reviewed articles on large prospective, retrospective and cohort studies that investigated obesity and unhealthy lifestyle habits as probable risk factors for the development of breast cancer. The selected articles concerned BRCA1 / 2 mutant women and investigated whether this status could increase the risk of breast cancer in relation to exposure to certain lifestyles. Studies that considered women without BRCA 1/2 mutations (in the case or control group), those with analyses that incorporated untested individuals or tested negative women, series with fewer than 100 patients enrolled, meta-analyses and reviews were excluded from this review (Fig. 1). One reviewer screened the titles and abstracts to select the studies and reviewed the full-text publications to confirm their eligibility and extract the relevant information from the included trials. A predefined spreadsheet (Excel 2007, Microsoft Corporation®) was used for data extraction. The most significant articles for lifestyles considered in this review are listed in Tables 1, 2, and 3.

**Table 1:** Article list used to analyzed cancer penetrance associated to weight gain and change body composition

***Authors/years***	***Reference***	***Study Design***	***Population***	***Sample Size***	***Control Group***	***Outcomes Measures***	***Main Conclusion***
***Bruno O. 2020***	*Clinical Breast Cancer*	*Cohort-Trial -Study*	*BRCA1/2 mutation carriers*	*502,0*	*yes*	*Change in body weight and composition and lifestyle characterisics*	*Higher fat mass associated with ncrease BC risk with greater effects in BRCA-2*
***Qian F. 2019***	*J Natl Cancer Inst*	*Randomized study*	*BRCA1/2 mutation carriers*	*11,451*	*yes*	*Anthropometric measures*	*Height and BMI associated to BC risk*
***Kim SJ. 2018***	*Int J of Epidem*	*Cohort-Study*	*BRCA1/2 mutation carriers*	*3734*	*yes*	*Changes in body weight*	*No association with breast cancer risk*
***Manders P. 2011***	*Br Can Res Treat*	*Cohort-Study*	*BRCA1/2 mutation carriers*	*719,0*	*yes*	*Anthropometric measures*	*Overweight and weight gain increase risk of postmenopausal breast cancer*
***Kotsopoulos J. 2005***	*Br Can Res*	*Multicenter study*	*BRCA1/2 mutation carriers*	*3291*	*yes*	*Changes in body weight*	*Weigh loss within 30 yrs reduces breast cancer risk while no association later in life*

**Table 2:** Article list used to analyzed cancer penetrance associated to harmful lifestyle habit

***Author/years***	***Reference***	***Study Design***	***Population***	***Sample Size***	***Control Group***	***Outcomes Measures***	***Main Conclusion***
***Li H. 2020***	*Cancer Epidemiol Biomarkers Prev*	*Cohort-study*	*BRCA1/2 mutation carriers*	*13,118*	*no*	*Alcohol consumption, smoking,*	*Smoking associated to BC risk for beginning to smoke more than 5 yers before full term pregnancy, no association with alcohol consumption*
***Kehm RD. 2020***	*Cancer Res*	*Cohort-study*	*BRCA1/2 mutation carriers*	*15,550*	*yes*	*Physical activity*	*Physical inactivity associated to 20% reduction BC risk*
***Ko KP. 2018***	*Int J Cancer*	*Longitudinal cohort study*	*BRCA1/2 mutation carriers*	*7195*	*no*	*Tobacco smoking*	*Tobacco smoking is associated with a modest increase of breast and ovarian cancer*
***van Erkelens A. 2017***	*J Genet Counsel*	*Cohort-Study*	*BRCA1/2 mutation carriers*	*268,0*	*no*	*Physical inactivity, smoking and alcohol consumption around childbearing age*	*Higher breast cancer risk in overweight women who drink alcohol, smoke and do not exercise*
***Dennis J. 2010***	*Breast*	*Case-control study*	*BRCA1/2 mutation carriers*	*1925*	*yes*	*Alcohol consumption*	*No association between alcohol consumption and BC risk*
***Ginsburg O. 2009***	*Breast Can Res Treat*	*Case-control study*	*BRCA1/2 mutation carriers*	*2538*	*yes*	*Smoking*	*Increase BC risk in BRCA 1 carriers with a past history of smoking*
***Mc Guire V. 2006***	*Cancer Epidemiol Biomarkers Prev*	*Case-control study*	*BRCA1/2 mutation carriers*	*323,0*	*yes*	*Alcohol consumption*	*No association between alcohol intake and BC risk*
***Ghardirian P. 2004***	*Int J Cancer*	*Case-control study*	*BRCA1/2 mutation carriers*	*1097*	*yes*	*Smoking*	*Smoking is not a risk factor for BC*

**Table 3:** Article list used to analyzed cancer penetrance associated to oral contraceptive use

***Authors/years)***	*Reference*	*Study Design*	*Population*	*Sample Size*	*Control Group*	*Outcomes Measures*	*Main Conclusion*
***Schrijver LH. 2018***	*JNCI Canc Spectr*	*Cohort Study*	*BRCA1/2 mutation carriers*	*9839*	*yes*	*Oral contraceptive use*	*No association between this use and BC risk*
***Lee E. 2008***	*Canc Epi Biom Prev*	*Population based study*	*BRCA1/2 mutation carriers*	*1469*	*yes*	*Oral contraceptive use*	*No association between this use and BC risk*
***Narod SA. 2002***	*J Cell Biol*	*Case control study*	*BRCA1/2 mutation carriers*	*1311*	*yes*	*Oral contraceptive use*	*Increase BC risk only in BRCA 1 women who used oral contraceptive before 30 years.*

## Results

Inheritance of a BRCA 1 or 2 mutation is associated with an increased lifetime risk of breast cancer [13]. The relationship between anthropometric characteristics such as weight gain and /or BMI and breast cancer risk has been examined extensively [14]. In 2014, a meta analysis selected 44 articles, according to established quality criteria, considering smoking and alcohol consumption as a risk factor for the onset of BC. The authors found that subjects who smoked for more than 4 years were at greater BC risk than those who had never smoked (ES = 1.97; 95% CI = 1.43 to 2.72) while no correlation was highlighted in works that investigated the habitual intake of alcohol vs total abstemia (ES = 0.87; 95% CI = 0.50 to 1.23) [15].

### Cancer penetrance associated to weight gain and changes in body composition

Weight gain and unfavorable changes in body composition with a significant increase in percentage body fat and decreased lean body mass are risk factors for breast cancer [16–19]. The relationship of anthropometric parameters and body weight changes with breast cancer risk has been examined extensively among women in the general population and several studies have investigated the impact of weight gain and cancer risk in women with a BRCA 1 or BRCA 2 mutation.

In a recent article of 2019, Qian F et al. investigated whether height or body mass index (BMI) could change the risk of developing breast cancer in 11,451 cases of breast cancer in BRCA 1/2 mutation carriers. These authors found that height was positively associated with breast cancer risk (per 10 cm increase HR = 1.9, 95% CI = 1.0 to 1.17; *p* = 1.17) while BMI was inversely associated with breast cancer risk (per 5 kg/ m^2^ increase HR = 0.94, 95% CI = 0.90 to 0.98; *p* = 0.007) [20].

In 2005, a multicenter study by Kotsopoulos J et al. investigated body weight changes and breast cancer risk in a total of 3291 women who carried BRCA 1 or BRCA 2 mutations and provided information on weight at ages 18, 30 and 40 showing that a weight loss of at least 4.5 kg between ages 18 and 30 was associated with a significant reduction in breast cancer risk (34%) thereafter (OR = 0.66; 95% CI 0.46–0.93). Weight gain later in life was not associated with increased risk [21]. In a multicenter longitudinal cohort study of 2018, Kim SJ et al. investigated the relationship between body size and breast cancer risk in 3734 BRCA mutation carriers and found no association between height, BMI and weight change and breast cancer risk [22]. In a retrospective cohort study published in 2011, Manders P. et al. investigated the association between anthropomentric measures and BC risk in 719,0 women with BRCA1 or BRCA2 mutation in pre and post menopause. The results reported in the work showed a decrease in risk in relation to BMI at 18 years while in postmenopausal age there was an increased risk respect to weight and in particular a higher BC risk in women weighing> 72 kg compared to those with weight < 72 kg, suggesting that postmenopausal mutated women should pay close attention to maintaining their body weight [23]. The summary list of these works is shown in Table 1

### Cancer penetrance associated to harmful lifestyle habits

Smoking, drinking alcoholic beverages [24, 25] and physical inactivity [26] are well-known lifestyle risk factors for pre- and postmenopausal breast cancer in the general female population. The association between breast cancer risk and these unhealthy lifestyle choices has been also investigated in BRCA 1/2 mutation carriers.

In a recent retrospective and prospective cohort study of 2020, Li H et al. investigated the association between smoking and alcohol consumption and the risk of developing breast cancer in 13,118 BRCA 1/2 mutation carriers and found that the only variable associated with the risk of BC for both carrier groups it was for mutated women who had smoked for at least 5 years before their first pregnancy compared to first-time mothers who had never smoked before. The results, found no correlation between BC cancer risk and alcohol intake in in both groups [27].

In 2020, Kehm RD et al. carried out a prospective cohort study on 15,550 women who had a familial breast cancer risk and investigated the association between recreational physical activity and decreased risk in adult women. The authors tested interactions of physical activity with predicted absolute familial BC risk based on BRCA 1 and BRCA 2 mutation status and concluded that physical activity can reduce the risk of getting BC by about 20% even women with high penetrance due to their genetic family history or medical history [28].

van Erkelens A et al. in a cohort study in 2017, investigated the correlation of unhealthy lifestyles (alcohol intake, smoking and low physical activity) in 268,0 women with BRCa 1 and 2 mutation reporting that 38% of the participants had at least 2 high risk factors for BC, plus age the diagnosis of the mutation correlated with a decrease in physical activity (OR = 0.93/year, 95% CI = 0.86–0.99) and a prevalence of overweight (OR = 1.07/year, 95% CI =1.02–1.13) [29].

Dennis J et al. in 2010 conducted a case-control study of 1925 premenopausal women who carried a BRCA 1 or BRCA 2 mutation to investigate alcohol consumption and the risk of breast cancer, reporting an inversely proportional association between alcohol intake and increased risk of developing BC only in BRCA1-mutated women, while no association was found in BRCA2-mutated women (OR = 0.82; 95% CI 0.70–0.96) vs OR = 1.00; CI 0.71–1.41) [30].

In 2004, Ghadirian P et al. studied the correlation between smoking and the risk of breast cancer in a large cohort of BRCA 1 and BRCA 2 mutation carriers [31]. The authors, in a case-control study conducted on 1097 BRCA1 and 2 mutated women vs healthy women, found no significant association between the 2 groups considered (mutated vs healthy women) whether they were smokers or ex smokers or the age at which smoking began within 5 years of menarche (OR = 1.03;95% CI = 0.90 to 1.33) or before the first pregnancy concluding that smoking could not be a breast cancer risk in carriers of BRCA mutations.

Two other eligible studies by Ko KP et al. in 2018 and Ginsburg O et al. in 2009 investigated the association between smoking and increased breast cancer risk. The first was a cohort study of 7195 women that demonstrated an increased risk of breast and ovarian cancer in women smokers with a BRCA 1 or BRCA 2 mutation (HR = 1.17; 95% CI 1.01–1.37), [32] and the second was a case control study of 2538 BRCA 1/2 carriers that showed a modest, but significant increase BC risk in BRCA 1 carriers with a past history of smoking (OR = 1.27; 95% CI 1.06–1.50) [33]. McGuire V et al. in 2006 investigated the association between alcohol consumption and increased risk of breast cancerin 323,0 women suggesting no positive association between alcohol intake and breast cancer risk in BRCA 1 and BRCA 2 mutation carriers aged [34]. The summary list of these works is shown in Table 2

### Cancer penetrance associated to oral contraceptive use

In the general population, multiparity and breastfeeding are among the protective factors of the risk of developing breast cancer, while the use of oral contraceptives could represent a predisposing factor; the data in the literature on the use of contraceptives in mutated women are still discordant but a possible role of estrogens on carcinogenesis has its foundation [35, 36]. The BRCA 1 /2 genes are involved in several functions including DNA damage and repair so the cancer-promoting effects of estrogen can be stronger in mutated BRCA 1 or BRCA 2 genes [37].

In 2018, Schrijver LH et al. investigated the association between the use of oral contraceptives and breast cancer risk in BRCA 1 and BRCA 2 mutation carriers in a retrospective and prospective cohort study of 9839 cases. They found no association between the use of oral contraceptives and BC risk in women with a BRCA 1 mutation (HR: =1.08, 95% CI = 0.75 to 1.56) while for BRCA 2 mutation carriers, the authors highlighted an increased risk of developing breast cancer in mutated women taking oral contraceptives (HR: =1.75, 95% CI = 1.03 to 2.97) and this risk was also related to the duration of treatment particularly in the period prior to the first full-term pregnancy [38].

In a 2008 population-based study on 1469 women and 444 control subjects Lee E et al. investigated reproductive factors and oral contraceptive use in BRCA 1/2 mutations carriers and non-carriers and reported no association between oral contraceptive use and BC risk in women carrying the mutations [39].

In 2002, in a matched case-control study on 1311 pairs of women with a known BRCA 1/2 mutation Narod SA et al. found an increased risk of breast cancer in women with a BRCA 1 mutation who first used oral contraceptives before age 30, or who used them for more than 5 years, while a similar risk did not appear in BRCA 2 mutation carriers [40]. The summary list of these works is shown in Table 3

## Discussion

**Fig. 2:**
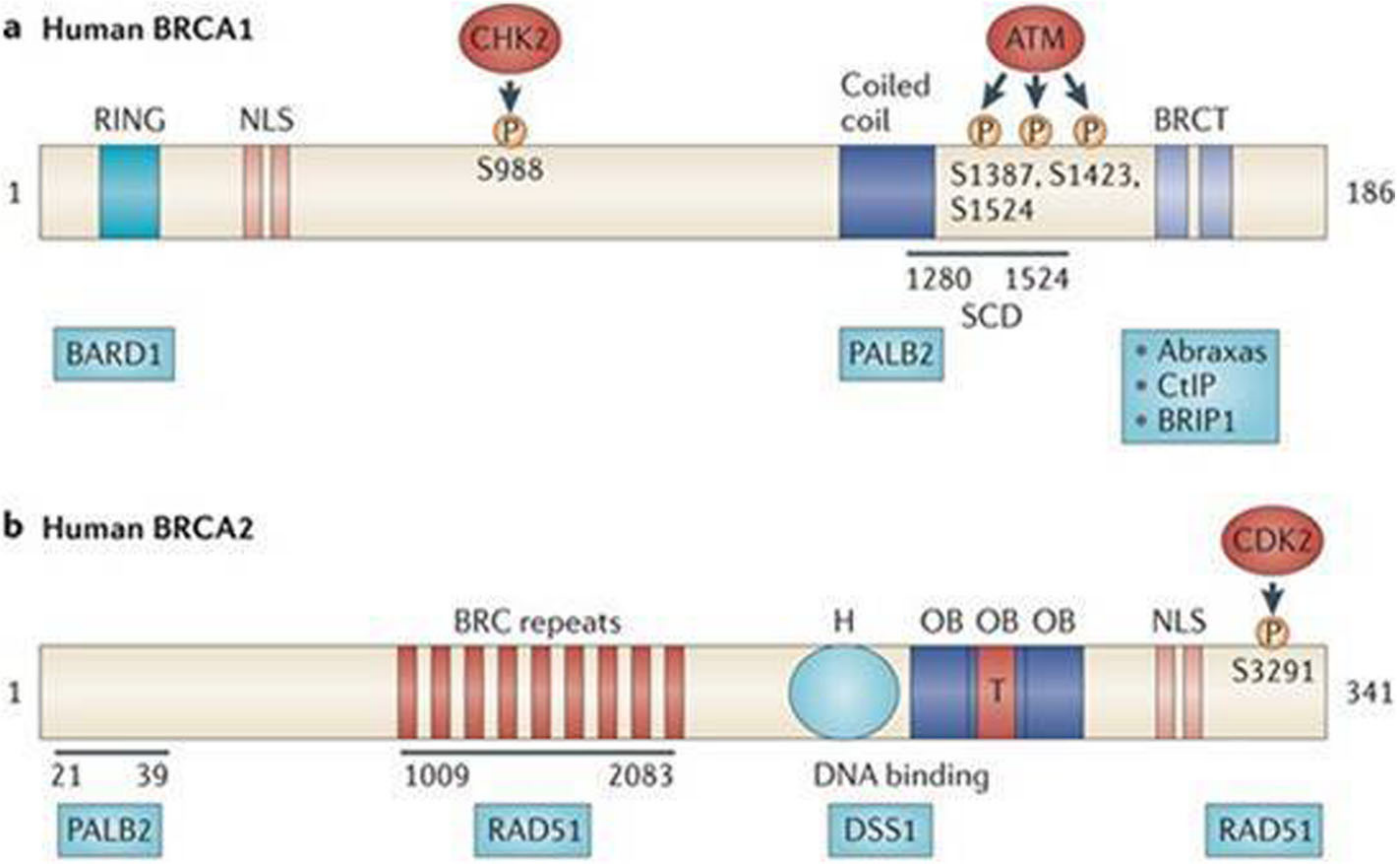
Chromosomal localization and structure of BRCA 1 and BRCA 2 genes. Image (idea from Narod SA et al. 2008)

The presence of BRCA1 and 2 mutations may predispose to a higher risk of breast cancer in the percentage of 60 and 30% respectively. It is important to underline that not all mutated women will certainly develop cancer during their lifetime, but knowing the genetic or environmental risk factors that can increase the subjective predisposition to cancer is of fundamental importance. It has been proposed that several lifestyle factors such as smoking, alcohol consumption, poor nutrition or sedentariness may be potential modulators of BRCA penetrance, but the data reported in the literature are still conflicting and incomplete. The BRCA 1 gene and the BRCA 2 gene located on chromosome 17 and on chromosome 13, respectively (Fig. 2), are tumor suppressors capable of regulating cell proliferation and repairing any damage in DNA replication. It is therefore plausible that carcinogens that are contained, for example, in cigarette smoke or in some food may increase the risk of BC in female carriers of BRCA 1/2 mutations. The carcinogens present in the cigarette have the ability to infiltrate the pulmonary alveolus [41] and the bloodstream flowing into the breast by means of plasma lipoproteins [42, 43]. They are lipophilic, tobacco-related carcinogens can be stored in breast adipose tissue [33, 34] and then metabolized and activated by human mammary epithelial cells [44]. Many authors have reported that cigarette smoke contains various substances harmful to the breast parenchyma highlighting the presence of p53 mutation in the breast parenchyma of smokers compared to non-smokers [45]. On the others hand, heterocyclic amines and acrylamides, foods rich in starch and cooked at high temperatures (e.g., grilled or overcooked meat) or rich in animal protein or milk [11] may be potentially more likely to promote the development of breast cancer and favor BRCA penetrance. Although several studies have largely reported concordant results about the correlation between unhealthy lifestyle habits and sustained weight gain over time and breast cancer risk in the general population, few studies with large series have been conducted on women carrying the BRCA 1 and 2 mutation. This mini review considered 16 studies (12 prospective and 4 retrospective) that highlighted a discrepancy between the effects of some unhealthy lifestyle factors in increasing the risk of breast cancer. Alcohol consumption was not observed to have a key role in the onset of breast cancer while smoking, weight gain and physical inactivity, especially in postmenopausal age, seem to increase the risk of breast cancer.

## Conclusion

Numerous factors have been reported to modify breast cancer risk. Our review of the specific literature has highlighted that there are few consistent results across different studies and that additional research would be beneficial to better determine the actual role and impact of such factors.

## Data Availability

Not applicable for that section.
